# Stress cardiac magnetic resonance imaging in elderly patients

**DOI:** 10.1186/1532-429X-13-S1-P102

**Published:** 2011-02-02

**Authors:** Golmehr Ashrafpoor, Susanna Prat-Gonzalez, Amir-Ali Fassa, Yannick Magliano, Alain Naïmi, Juan Sztajzel

**Affiliations:** 1 Cardiology Service, Geneva University Hospitals, Geneva, Switzerland; 2 Centre de Diagnostic Radiologique de Carouge, Geneva, Switzerland

## Introduction

The prevalence of coronary artery disease increases with age. A significant proportion of patients commonly referred for investigation of coronary artery disease are often unable to perform exercise testing because of advanced age, and can only be assessed with pharmacologic tests. Stress cardiac magnetic resonance (CMR) imaging is a non-invasive modality used for detection of myocardial ischemia, necrosis and viability.

## Purpose

The aim of the present study is to determine the applicability and safety of stress CMR in patients older than 70 years.

## Methods

We reviewed the data of all patients older than 70 years who were referred for stress CMR (1.5 Tesla) from January 2006 to February 2010 to our outpatient center. Standard protocol consisted of: 1) assessment of myocardial function at rest; 2) pharmacological stress induced either by dobutamine (protocol of 10, 20, 30, 40 µg/kg/minute during 3 minutes with atropine if necessary) until achieving submaximal heart rate ([220-age] x 0.85), or by adenosine (protocol of 140 µg/kg/minute during 3 minutes followed by a bolus of 10 ml of gadolinium at 4 ml/second "first pass"); 3) assessment of myocardial scar and/or viability by delayed enhancement sequences.

## Results

Among the 309 patients ≥70 years old referred for a stress CMR, the test could be performed in 297 (96%) patients (172 [57%] males, mean age 76±4 years [range 70-91 years]). Mean test duration was 50±9 minutes. Phamacological stress was induced with dobutamine in 98 (33 %) patients and adenosine in 199 (67 %) patients). The test could not be carried out in 12 (4 %) patients because of claustrophobia (8 patients) and excessive thoracic diameter (4 patients). Among the patients who underwent stress CMR, target heart rate was not reached in 13 (4%) patients. Side effects included one case of sustained supraventricular tachycardia and one case of a transient severe hypotension. In 4 patients gadolinium contrast was not injected due to severe renal insufficiency. No other complications occurred. No ischemia or infarction was found in 170 patients (58%), while isolated ischemia was found in 20 patients (7%) and ischemia in the presence of an infarction in 34 patients (12 %). Infarction without ischemia was found in 67 patients (23%).

## Conclusions

Our data shows that stress CMR performed in elderly ambulatory patients is safe and well tolerated. Myocardial ischemia and/or infarction could be confirmed in 42% and excluded in 58% of patients in less than one hour exam.

